# Transcanalicular laser dacryocystorhinostomy for acquired nasolacrimal duct obstruction: an audit of 104 patients

**DOI:** 10.1186/s40001-018-0355-4

**Published:** 2018-11-16

**Authors:** Joel M. Mor, Mario Matthaei, Holger Schrumpf, Konrad R. Koch, Edwin Bölke, Ludwig M. Heindl

**Affiliations:** 1 Department of Ophthalmology, University of Cologne, Kerpener Strasse 62, 50924 Cologne, Germany; 2 Department of Dermatology, University of Dusseldorf, Düsseldorf, Germany; 3 Department of Radiation Oncology, University of Dusseldorf, Düsseldorf, Germany

**Keywords:** Nasolacrimal duct obstruction, Laser, Dacryocystorhinostomy, Transcanalicular, PANDO

## Abstract

**Purpose:**

External dacryocystorhinostomy (DCR) is considered as the gold standard in the treatment of acquired nasolacrimal duct obstruction. However, many advances have been made towards the development of modern minimally invasive therapies. These new techniques were proven less harmful to the patients’ skin and medial palpebral structures with their palpebral-canalicular pump mechanism. Options include endonasal and transcanalicular procedures. Here, we report on our 2-year experience with the surgical technique, results and complications of transcanalicular laser-assisted DCR.

**Methods:**

This is a retrospective study. A total of 104 patients with acquired nasolacrimal duct obstruction underwent transcanalicular laser-assisted DCR combined with bicanalicular silicon intubation. We then analyzed intra-/post-operative complications and subjective and objective success rates. The institutional ethics committee ruled that approval was not necessary. The trial was registered with the German Clinical Trials Register (DRKS00012879).

**Results:**

Transcanalicular laser-assisted DCR in combination with bicanalicular silicon intubation could be performed surgically successfully in 101 patients (97%). In three cases (3%) using the superior canalicular approach, positioning of the laser instrument at the anteroinferior rim of the middle turbinate failed. Complications included thermal injury to the canaliculus (one), canalicular infection (two) and silicon tube prolapse (ten). Functional success (resolution of preoperative symptoms) was achieved in 80 cases (77%), functional failure occured in 24 cases with all patients reporting persisting epiphora, 15 reporting failure to irrigate the nasolacrimal duct and 15 requiring secondary external DCR.

**Conclusions:**

Laser-assisted DCR shows promising results with few complications. It seems well suited as a second-step procedure after failed recanalization and before external DCR.

## Introduction

**Fig. 1:**
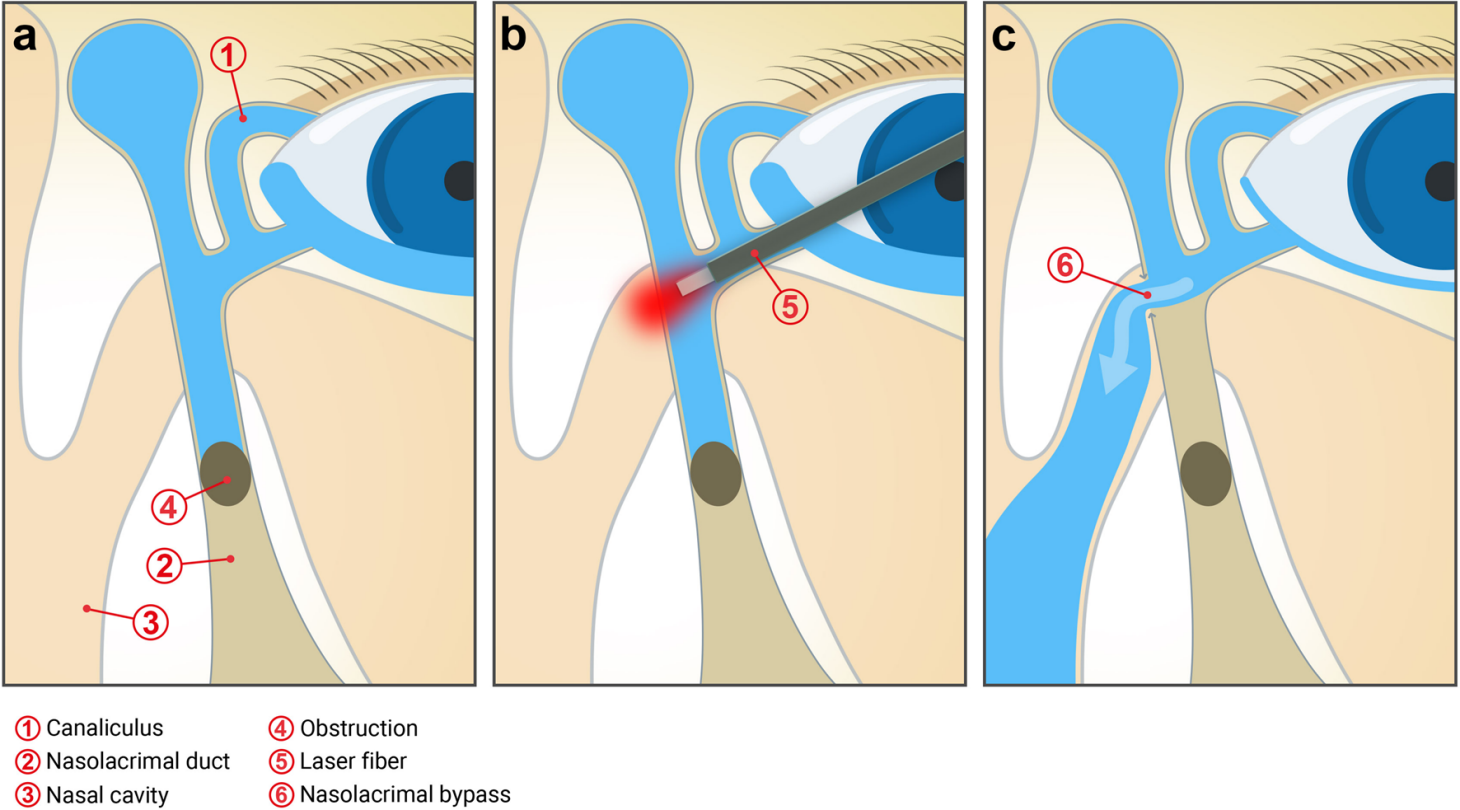
Schematic display of primary acquired nasolacrimal duct obstruction treated by laser-assisted dacryocystorhinostomy. **a** An infrasaccal obstruction of the nasolacrimal duct leads to epiphora. The canaliculi are unobstructed. **b** A laser fiber is positioned in the lacrimal sac, aiming at the nasal wall to create a nasolacrimal bypass. **c** After the ostium has been created, tear flow is redirected through the newly formed ostium into the nasal cavity, bypassing the nasolacrimal duct obstruction

External dacryocystorhinostomy (DCR), a procedure dating back more than a 100 years to when it was first performed by Addeo Toti [1], has for a long time been considered to be the gold standard in the surgical treatment of infrasaccal primary acquired nasolacrimal duct obstruction (PANDO; Fig. 1a). After incision of the skin and preparation of the lateral nasal wall at the height of the lacrimal fossa, a bony ostium is created using a drill. Subsequently, nasal mucosa and lacrimal sac mucosa are anastomosed, thus creating a generous bypass ensuring adequate tear drainage. However old the technique, success rates range between 82.7 and 94.1% [2, 3]. In the past years, endonasal DCR has gained popularity in some oculoplastic centers as well. Here, successful outcome ranges between 79 and > 90% depending on the study [4–8].

In recent years, the advent of advanced endoscopy technologies allowed for a handful of minimally invasive operating techniques to emerge, posing alternative treatment options. Two of which in particular, namely laser-assisted dacryoplasty and microdrill dacryoplasty, have been shown to be viable first-step procedures in the treatment of short-segment membranous stenoses of the nasolacrimal duct [9–11]. Not only do these procedures take less time than external DCR, but also treated patients show shorter convalescence [12–14].

Much effort has been put into closing the gap between the above-mentioned first-line procedures and external DCR as a definitive method. Likely the most promising approach, laser-assisted DCR has been reported to deliver successful results in 74–85% of cases depending on a variety of factors, e.g., patient age, use of silicon intubation and, arguably, mitomycin C (MMC) treatment. However, long-term results remain yet to be investigated. Laser-assisted DCR is performed by placing a small diode laser fiber in the lacrimal sac (Fig. 1b, c) and creating a nasolacrimal bypass in the medial saccal wall, i.e., the lateral nasal wall, by vaporization of the bone using laser energy. The instruments are inserted through either canaliculus and require no skin incision while visual control is performed endoscopically from the nasal cavity [15–22].

In this audit of 104 patients, we are giving an update on our 2-year experience in technique and complications of laser-assisted DCR in the treatment of infrasaccal PANDO [16].

## Subjects and methods

Between September 2013 and February 2016, laser-assisted DCR was performed in a total of 104 consecutive cases of acquired absolute postsaccal nasolacrimal duct obstruction, posing as an alternative to classic external DCR. We included patients with PANDO and did not offer this treatment option to those patients that had had previous lacrimal duct or nasal surgery. Tab. 1 displays a complete list of exclusion criteria.

**Table 1:** Exclusion criteria

Exclusion criteria
Congenital obstruction
Neoplastic obstruction
Traumatic obstruction
Canalicular stenosis
Intrasaccal stenosis
Dacryocystocele
Lid anomalies/malposition
Underlying rhinological disease
Prior nasolacrimal surgery

If patient history revealed one or more matches in these categories, patients were excluded from the study

Perioperative diagnostics and treatment as well as surgery itself were conducted as previously described [16]. Before surgery, patient history regarding severity (mild/moderate/severe), duration and type of symptoms (e.g., epiphora, swelling of the lacrimal sac, clotted eyelids, secretion of fluid, mucus, pus or blood) as well as history of previous episodes of dacryocystitis was taken. Next, patients were examined ophthalmically with particular regard to any possible superficial ocular lesions and/or infections and to the eyelids’ and tear dots’ position to identify cases of ectropium causing epiphora. In a standardized fashion, irrigation and probing of the lacrimal drainage system were performed using a Bangerter probe to identify location (presaccal vs infrasaccal) and extent (relative vs. absolute) of stenoses. In case of absolute infrasaccal nasolacrimal duct obstruction, the inserted probe was easily able to reach the wall of the lacrimal bone without the occurence of any bouncy resistance (“hard stop“), yet upon irrigation through the lower tear dot, contralateral reflux occured out of the upper tear dot and vice versa, while no fluid was able to pass the nasolacrimal duct. Additionally, an examination by an otorhinolaryngologist including endoscopic visualization of the nasal cavity was carried out to rule out intranasal pathologies, e.g., benign or malignant tumors, nasal septum deviation or anatomical anomalies of the turbinates that could potentially compromise the procedure and the functional outcome.

**Fig. 2:**
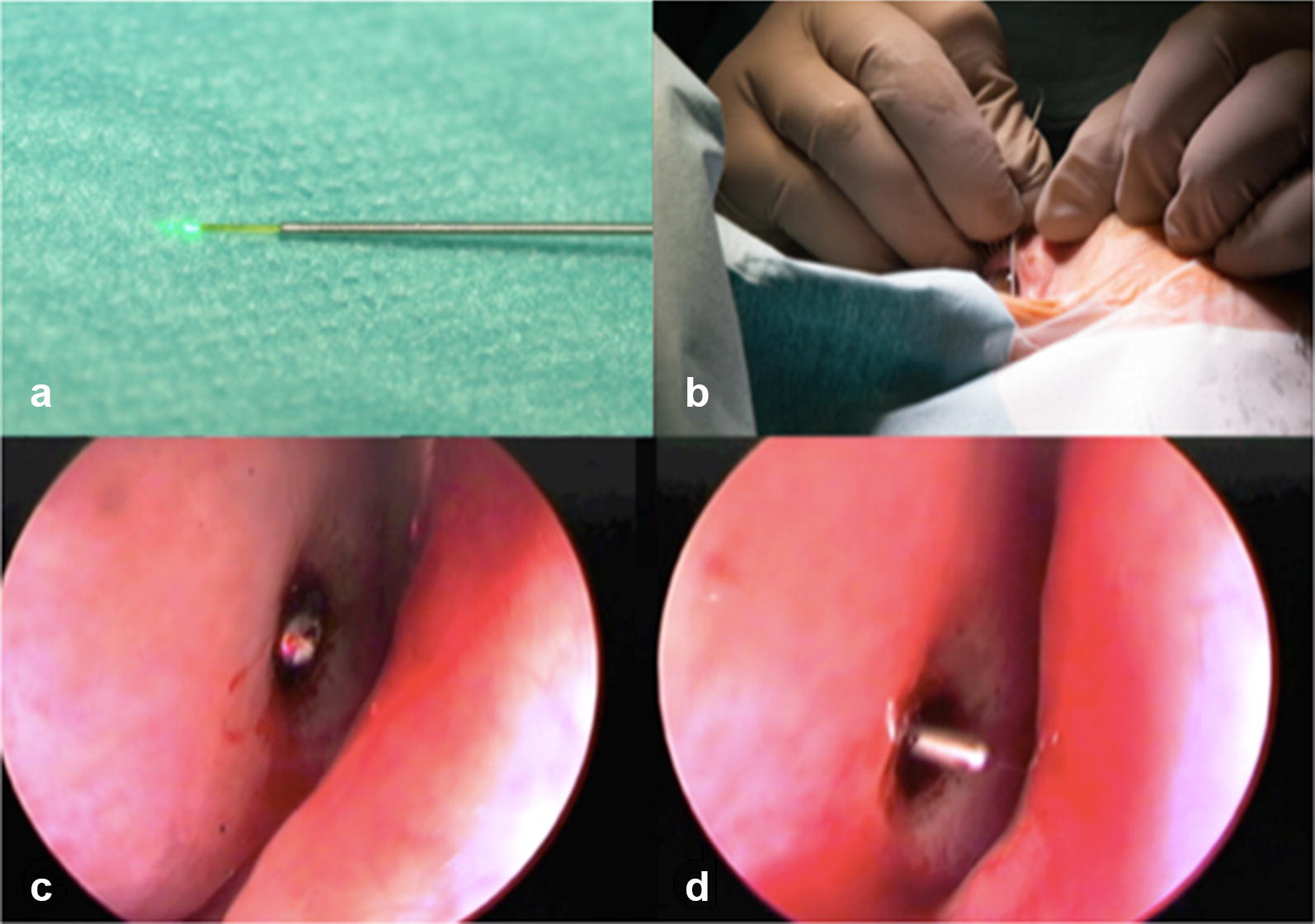
Laser-assisted dacryocystorhinostomy. **a** Laser fiber (300 µm in diameter), connected to a 810-nm wavelength diode laser, fitted into a handpiece. **b** Correct positioning of the laser fiber with mediorostral orientation. **c** Transillumination shortly before the tip breaks through the nasal mucosa at the anteroinferior rim of the middle turbinate. **d** A blunt metal probe guiding a silicon tube is pushed through the ostium

For transcanalicular laser-assisted DCR, patients were put under general anesthesia. Firstly, using endonasal endoscopy, the anterior margin of the middle turbinate was visualized. A laser fiber optic (300 µm in diameter), connected to a 810-nm wavelength diode laser (Fox; A.R.C. Laser GmbH, Nürnberg), was fitted into a handpiece for adequate maneuvering as well as a blunt tear duct cannula, letting 2–3 mm of the fiber optic stick out at the tip of the cannula (Fig. 2a). To prevent unnecessary thermal injury to the surrounding tissues upon initial activation of the laser, the tip was first carbonized by holding it on a wooden spatula [23, 24]. After bouginage of either tear dot, the fiber was inserted and carefully pushed into the lacrimal sac. Aiming the tip in an anterior inferior direction, constant contact to the bone wall of the lacrimal sac was kept. For the first 20 patients, the upper tear dot along with the upper canaliculus was chosen as an entry to the tear ducts. However, since we often experienced difficulties in the correct positioning of the laser because of an overly prominent orbital rim anatomy, we then shifted to using the approach via the lower tear dot. Positioning of the laser fiber was corrected under visual control via nasal endoscopy (Fig. 2b, c). Here, the laser’s aiming beam could be detected and fine adjustments were made until it appeared at the anterior inferior rim of the base of the middle turbinate. Next, laser energy was applied (power 5–10 W, pulse duration 90 ms, exposition pause 50 ms). Keeping contact to the wall, we were careful not to apply too much pressure in order not to thermally damage any surrounding saccal mucosa or push the tip of the laser fiber back into the cannula, which in turn can result in heating and thermal injury as well. Upon breaching the wall and, thus, creating the required bony ostium, we enlarged said ostium in a circular manner by vaporizing the margins with further laser spots, the goal being a diameter of 5 mm (Fig. 2c, d). Successful anastomosis was confirmed by successful irrigation with saline. In contrast to other authors, we did not apply mitomycin C (MMC) [22] to the new-formed anastomosis. Finally, monocanalicular silicon tubes were placed in both canaliculi (Wide Collarette Monoka^®^; Fa. FCL, Paris, France) and led into the nasal cavity through the artificial ostium.

Postoperative treatment consisted of xylometazolin 0.05% eyedrops to help the swelling, steroidal (prednisolon) and antibiotic (ofloxacin) eye drops to prevent inflammation and infection. While antibiotics were stopped after 1 week, steroidal and decongestant eye drops were slowly weaned off over the course of 4 weeks.

Clinical follow-up was at 6 weeks, 3 months and 6 months after surgery. The silicon tubes were removed 3 months after surgery. Surgical success was defined as there being a patent osteotomy between nasolacrimal sac and nasal cavity as well as successful bicanalicular silicon intubation. Functional success was defined as complete resolution of symptoms at least 6 months after surgery. Failure was defined as either missing melioration (postoperative epiphora, recurring dacryocystitis), impossibility of irrigation after up to 6 months or necessity of revision surgery.

For statistical analysis, SPSS Software (Windows version 21.0; SPSS, Inc., Chicago, IL, USA) was used.

## Results

Laser-assisted transcanalicular DCR was performed in 104 patients. An overview of epidemiological data can be found in Table 2. All patients were suffering from severe symptoms preoperatively: epiphora in 98%, clotted eyelids in 67%, mucopurulent tear discharge in 48%, and erythematous swelling of the lacrimal sac in 5%. In 3% of cases, patients reported episodes of acute dacryocystitis in the past (dating back 3 weeks in one case and more than 6 months in two cases).

**Table 2:** Overview of epidemiological data

Epidemiological data	
Total number of patients	104 (100%)
Male	27 (26%)
Female	77 (74%)
Mean age at time of surgery	59 ± 10 years
Right side	60 (58%)
Left side	44 (42%)
Upper punctum approach	20 (19%)
Lower punctum approach	84 (81%)
Surgical success	101 (97%)
Surgical failure	3 (3%)
Functional success	80 (77%)
Functional failure	24 (23%)

Surgical success was defined as ostium patency following laser-assisted dacryocystorhinostomy with bicanalicular silicon intubation. Functional success was defined as complete resolution of symptoms at 6 months after surgery

As per our aforementioned definition, laser-assisted DCR with subsequent bicanalicular silicon intubation was performed surgically successfully in 101 cases (97%). For the first 20 patients, we chose the upper tear dot and canaliculus for entry. However, in three of these cases (3%), optimal positioning of the diode laser fiber proved mechanically impossible due to prominence of the upper orbital rim. Two of which received a patent osteotomy at the back of the middle turbinate without subsequent silicon intubation. In the third case, coincident tight intranasal conditions required conversion into external DCR. As previously described [16], we then switched to taking the approach via the lower tear dot. Notably, all further 84 cases were surgically successful and correct positioning of the equipment was possible in all 84 cases.

**Fig. 3:**
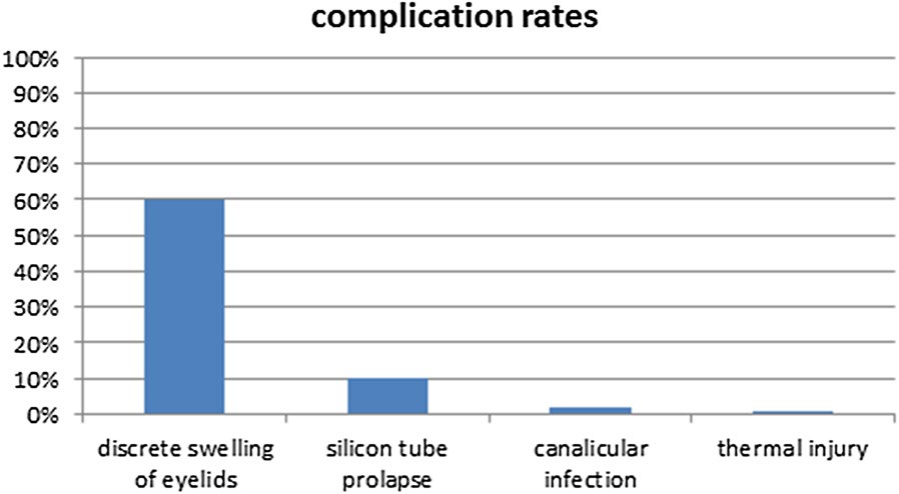
Complication rates (%) following laser-assisted dacryocystorhinostomy

Postoperative complications were generally mild, severe complications occured only rarely (Fig. 3). Most commonly, 62 patients (60%) developed discrete swelling of the eyelids for 1–2 days. Ten patients (10%) had a silicon tube prolapse, two (2%) developed canalicular infection (one of which being the patient that had reported an episode of acute dacryocystitis 3 weeks prior to surgery). Thermal injury to the surrounding tissue with subsequent necrosis occured in one case (1%) and had to be treated by canaliculus suture and a small displacement flap.

Functional success 6 months after the treatment was achieved in 80 cases (77%); functional failure with a relapse of symptoms occured in 24 cases (23%). Of the latter, all 24 suffered from postoperative epiphora, 15 did not have a patent ostium and a total of 15 patients required secondary external DCR (one of these being one of the two patients that had received the osteotomy at back of the middle turbinate). A relationship between the chosen approach (upper vs. lower canaliculus) and the effectiveness of the procedure could not be shown.

## Discussion

In this study, we are reporting on our 2-year experience in laser-assisted DCR treatment for infrasaccal PANDO. The procedure (performed as described above) is showing promising results with a surgical success rate of 97% and functional success rate of 77% after 6 months. Postoperative complications were mild, discrete swelling of the eyelids being the most common one at 60% of cases. Functional failure 6 months after surgery occured in 23% of patients.

These results are similar to those of other publications with success rates generally ranging from 74 to 85% [15–22]. To improve results, additional steps are often taken. As is common in the majority of protocols, we used silicon intubation after creating an osteotomy [15–21]; yet, this step is still the subject of debate and solid evidence of the assumed benefit remains to be delivered. In a recent study comparing patients undergoing endonasal DCR with and without silicon intubation, a significant benefit could be shown for the group receiving silicon intubation [8]. This allows the assumption that the benefit of silicon intubation might, in part, depend on the surgical procedure chosen [8]. Additionally, some authors apply the anti-metabolite MMC after creating the osteotomy to further inhibit scarring. However, recent studies have shown no significant difference in success rates between patients treated with and without MMC, respectively [22, 25].

The current gold standard, external DCR, provides functional success rates above 82–90% depending on the study [2, 3, 15, 18]. Several possible reasons for its slightly higher effectiveness compared to laser-assisted DCR have to be debated. Firstly, in external DCR, the osteotomy created by the drill is bigger than those created in any other procedure including endonasal DCR. Obviously, the bigger the osteotomy, the less likely it is to be obstructed by newly formed scar tissue postoperatively.

As an alternative to external DCR, some oculoplastic centers perform endonasal DCR of various types as primary treatment for PANDO, yielding mixed results depending on the center and the method performed. Here, functional success rates ranging from 79% to above 90% have been reported [4–8].

One major advantage of external DCR is the opportunity to fully examine the lacrimal sac and, in case of suspected secondary obstruction (atypical findings, e.g., granulomatous or neoplastic disease), take a sample for further analysis [26]. Likewise, endonasal DCR allows for biopsies to be taken where needed. In these cases, either external or endonasal DCR is unavoidable.

Even though functional success rates are higher for external DCR (and, partly, endonasal DCR) [2, 3, 15, 18] than for laser-assisted transcanalicular DCR [15–22], the reported difference is not enormous and several advantages that the minimally invasive procedure has over its invasive counterpart have to be taken into account. Firstly, about 3% of patients, mostly younger ones, have been reported to be unhappy with the cosmetic outcome of external DCR. By not having to make a skin incision along the back of the nose, scarring of the affected region can be avoided, thus adding to patient satisfaction. Secondly, postoperative convalescence is quicker [14, 27]. Thirdly, the duration of surgery is lower in laser-assisted DCR, taking only 10–25 min, while external DCR usually takes between 35 and 75 min [13, 16–18, 22]. Both procedures usually require general anesthesia, however. Finally and most importantly, the minimally invasive procedure is able to spare the anatomical structures of the medial lid including the medial canthal tendon and the part of the Horner’s muscle that stretches to the lacrimal sac. These structures are essential for the function of the physiological lacrimal pump mechanism that ensures adequate tear passage. This is evidenced by the fact that after external DCR, even if there is a patent, irrigatable ostium, delayed filling of the lacrimal sac can be observed [28, 29].

Nevertheless, laser-assisted DCR is not without its drawbacks. For one thing, the total amount of laser energy applied should be kept to the minimum required as over-exposure can cause scarring, thus leading to secondary occlusion. Possibly, the application of heat, in particular, as a means of vaporizing the bone might provoke the formation of granulation tissue that has been accused of being responsible for secondary obstruction [30]. Unfortunately, precise specifications on laser energy, pulse duration and pause duration have not yet been defined. Also, there is a certain risk of thermal injury. In our study, one patient suffered from tissue necrosis with the formation of a cutaneous fistula after the laser fiber’s tip shifted. It is crucial that the tip stays in place with a distance of at least 2–3 mm from the blunt metal cannula. Otherwise, if the tip is accidentally retracted into the cannula, it can cause heating of the metal and damage the surrounding organs. Similarly, application of too much lateral pressure on the tip can cause it to break off. This can also result in heating of the cannula.

## Conclusions

In conclusion, laser-assisted DCR is showing promising success rates in the treatment for infrasaccal PANDO that are close to those of external DCR, while at the same time having the advantage of being minimally invasive. The procedure can be performed relatively quickly, all the while sparing skin and medial lid structures, thus protecting the physiological lacrimal pump mechanism. Furthermore, even in case of failure, external DCR is still an option. Therefore, laser-assisted DCR is a viable option serving as a “second-step procedure” to close the gap between recanalizing procedures and external DCR.
